# Targeting CaN/NFAT in Alzheimer’s brain degeneration

**DOI:** 10.3389/fimmu.2023.1281882

**Published:** 2023-11-23

**Authors:** Joanna Mackiewicz, Malwina Lisek, Tomasz Boczek

**Affiliations:** Department of Molecular Neurochemistry, Medical University of Lodz, Lodz, Poland

**Keywords:** calcineurin, NFAT, Alzheimer’s disease, calcium, inflammation

## Abstract

Alzheimer’s disease (AD) is a neurodegenerative disorder characterized by a progressive loss of cognitive functions. While the exact causes of this debilitating disorder remain elusive, numerous investigations have characterized its two core pathologies: the presence of β-amyloid plaques and tau tangles. Additionally, multiple studies of postmortem brain tissue, as well as results from AD preclinical models, have consistently demonstrated the presence of a sustained inflammatory response. As the persistent immune response is associated with neurodegeneration, it became clear that it may also exacerbate other AD pathologies, providing a link between the initial deposition of β-amyloid plaques and the later development of neurofibrillary tangles. Initially discovered in T cells, the nuclear factor of activated T-cells (NFAT) is one of the main transcription factors driving the expression of inflammatory genes and thus regulating immune responses. NFAT-dependent production of inflammatory mediators is controlled by Ca^2+^-dependent protein phosphatase calcineurin (CaN), which dephosphorylates NFAT and promotes its transcriptional activity. A substantial body of evidence has demonstrated that aberrant CaN/NFAT signaling is linked to several pathologies observed in AD, including neuronal apoptosis, synaptic deficits, and glia activation. In view of this, the role of NFAT isoforms in AD has been linked to disease progression at different stages, some of which are paralleled to diminished cognitive status. The use of classical inhibitors of CaN/NFAT signaling, such as tacrolimus or cyclosporine, or adeno-associated viruses to specifically inhibit astrocytic NFAT activation, has alleviated some symptoms of AD by diminishing β-amyloid neurotoxicity and neuroinflammation. In this article, we discuss the recent findings related to the contribution of CaN/NFAT signaling to the progression of AD and highlight the possible benefits of targeting this pathway in AD treatment.

## Introduction

Alzheimer’s disease (AD) is a chronic neurodegenerative disorder characterized by a progressive decline in cognitive and executive functions (1). Intensive research performed over the decades has revealed the complex mechanistic underpinnings of AD, involving a combination of age-dependent brain changes along with genetic predisposition, lifestyle, and environmental factors (1). Despite relatively wide knowledge about its etiology, the cellular determinants of AD susceptibility or resilience and the key molecular changes driving disease progression are less understood.

Neuropathologically, AD-associated degeneration is linked to the deposition of amyloid-β (Aβ) fibrillary aggregates, occurring as neuritic plaques or vascular deposits, and hyperphosphorylated microtubule-associated protein tau. The latter is a major component of neurofibrillary tangles, neuropil threads, and neuritic plaque corona (2). However, the list of detected abnormalities is much longer and involves gliosis, neuroinflammation, oxidative stress, changes in neuronal plasticity, altered expression of glutamate and cholinergic receptors, Ca^2+^ homeostasis imbalance, and many others. The accumulation of these changes across the lifespan leads to impaired cognition and a higher risk of AD development. It is now well-accepted that many of these pathologies are a consequence of Ca^2+^ signaling deregulation, which is central to amyloid-evoked degeneration (3). Indeed, numerous independent biochemical, behavioral, electrophysiological, and molecular studies, which have been the subject of excellent reviews (1, 4, 5), have confirmed a link between Ca^2+^ deregulation and age-related memory deficits and worsening cognitive performance. Nonetheless, it is becoming apparent that Ca^2+^ imbalance is not restricted to neurons but also underlies the altered function of other non-neuronal cells, especially astrocytes. The relationship between astrocytic Ca^2+^ deregulation as a function of neurodegenerative diseases has been thoroughly discussed in comprehensive reviews (6–8). Several laboratories have provided strong evidence to imply that Ca^2+^-dependent protein phosphatase calcineurin (CaN) links dysfunctional Ca^2+^ signaling to Aβ accumulation, neuroinflammation, and synaptotoxicity (3, 9–11). The activity of CaN is controlled by spatially and temporarily restricted intracellular Ca^2+^ elevations, which activate specific Ca^2+^ signal decoding proteins (12). CaN is required for both long-term potentiation and long-term depression, synaptic processes underlying learning and memory, and its activity positively correlated with cognitive loss in AD brains (13, 14). Perhaps, one of the most important downstream effectors linking Ca^2+^/CaN signaling to the gene regulatory machinery is the nuclear factor of activated T-cells (NFAT). Mounting evidence, as will be discussed in the following sections, shows that CaN/NFAT drives or exacerbates the core symptoms of AD neuropathology. It is therefore critical to understand the upstream and downstream signals leading to the pathological activation of CaN/NFAT pathway to identify new therapeutic targets and develop new treatment strategies for AD. In this review, we summarize the emerging evidence that deficient CaN/NFAT could contribute to brain degeneration in AD.

## Calcineurin

**Figure 1:**
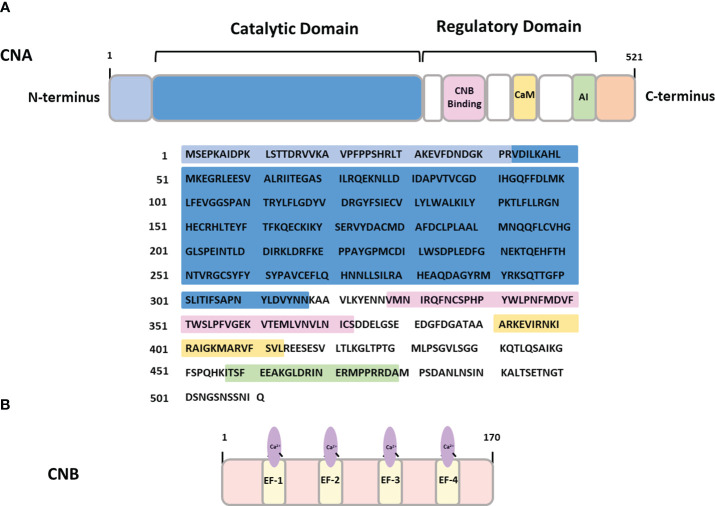
The structure of calcineurin **(A)** CNA- calcineurin subunit A containing binding sites for calcineurin subunit B (CNB binding), calmodulin binding domain (CaM) and autoinhibitory domain (AI). **(B)** CNB – calcineurin subunit B containing four Ca^2+^-binding EF hand domains.

In healthy adults, CaN is widely expressed throughout the brain, with its highest concentration detected in neurons and low in glial cells (15–18). Structurally, the holoenzyme of CaN comprises two main subunits: a ~ 60 kDa catalytic subunit (CNA) and a ~19 kDa regulatory subunit (CNB) containing four Ca^2+^-binding EF-hand motifs (12) (**Figure 1**).

Binding of calmodulin (CaM) to the regulatory domain of the catalytic subunit increases CaN phosphatase activity, decoding the upstream Ca^2+^ signals and translating them into a dephosphorylation pattern of cellular proteins. CaM itself is a small, evolutionary conserved protein expressed in all mammalian cells. It serves as a versatile Ca^2+^ sensor capable of responding to a wide range of Ca^2+^ concentrations (10^-12^ M - 10^-16^ M) due to the presence of four canonical Ca^2+^ binding EF hands possessing different affinities for Ca^2+^ (19). Upon binding Ca^2+^, CaM undergoes a conformational change exposing the hydrophobic surface region for Ca^2+^-dependent interaction with CaM-binding proteins (20). The Ca^2+^/CaM complex interacts with targets that mediate crucial cellular processes such as inflammation, metabolism, apoptosis, short-term and long-term memory and the immune response (21–25). Continued research indicates that CaM binds to and affects many proteins involved in the onset and progression of AD and other neurodegenerative diseases (26–28). This involvement in neurodegeneration underscores the significance of CaM in the understanding the molecular mechanisms behind these diseases.

Among many Ca^2+^-dependent protein phosphatases, CaN is the only one directly activated by Ca^2+^/CaM and is one of the most sensitive enzymes responding to Ca^2+^ elevations. The cooperative interaction between Ca^2+^, CaNB, and Ca^2+^/CaM allows CaN to uniquely respond to discrete Ca^2+^ fluctuations (29). These features make CaN particularly vulnerable to alterations in Ca^2+^ homeostasis observed in AD. A growing body of evidence suggests that upregulation of CaN activity is directly linked to multiple neurodegenerative insults observed in Parkinson’s disease (30), Alzheimer’s disease (31), and Huntington’s disease (32), all marked by impaired synaptic function, neuroinflammation, and neuronal loss. Moreover, abnormal activation of CaN has been observed in numerous cellular events traditionally linked to AD, including astrocyte activation, Aβ generation, neuronal apoptosis, synaptic toxicity, and behavioral deficits (14). The involvement of CaN is also supported by several studies showing altered CaN signaling in the brains of animals used to model the pathogenesis of neurodegenerative disorders (33–36).

Strong evidence for the contribution of CaN to the pathophysiology of AD in humans comes from the Taglialatela group (37). The results of a retrospective analysis of kidney transplant patients demonstrated that the administration of CaN inhbitors to prevent transplant rejection decreased the incidence of dementia and AD in this group compared to national data from the general population. The initially encouraging results were potentially limited by the small sample size. These findings have been recently replicated in a large cohort by Silva and colleagues (38), demonstrating that FK506, CsA, and sirolimus can reduce the risk of dementia compared to general population-like control. However, among the three immunosuppressive drugs, those that are capable of crossing the blood-brain barrier, like FK506, have a greater probability of reducing dementia. Since inflammatory mediators are strongly correlated with the accumulation of Aβ and induction of neuronal apoptosis, the potential role of CaN inhibitors in reducing the prevalence of dementia is not surprising. However, the study performed by Silva and colleagues delineates a new therapeutic avenue based on brain-penetrant CaN inhibitors. In addition, it has been shown that CaN is heavily involved in the regulation of gene expression (39). For upstream and downstream regulatory mechanisms of CaN, we refer to the excellent reviews published recently (12, 40–42).

## NFAT – structure, function and regulation

Proteins belonging to the NFAT family were originally described more than three decades ago as the transcriptional activators of interleukin-2 (IL-2) in T cells (43). It is now apparent that NFAT proteins play a key role not only during T-cell activation and differentiation but also in regulating the function of several types of immune cells, including B cells, mast cells, basophils, and natural killer cells (44). NFAT family members are involved in the induction and/or coordination of the immune response by modulating the expression of a large number of immunologically important genes. These genes include cytokines such as IL-2, IL-3, IL-4, IL-5, IL-8, IL-13, granulocyte-macrophage colony-stimulating factor (GM-CSF), and tumor necrosis factor-alpha (TNF-α), as well as cell-surface receptors CD40L and CTLA-4, and the apoptotic factor FasL (45).

NFAT family members are widely distributed throughout tissues, including the brain, and can regulate distinct developmental processes (46–49). The strong correlation between NFAT expression and vertebrate development was derived from mouse genetic experiments. For instance, deletion of NFATc1 caused dramatic defects in cardiac morphogenesis (50), NFATc2 null mice displayed abnormalities in chondrogenesis (51), disruption of NFATc3 resulted in dysregulation of myogenesis (52), whereas NFATc4 deficient mice were healthy and developmentally normal. Suchting et al. (53) have discussed the developmental relationship between nerve cells and vessels, pointing out several anatomical and functional parallels. In support of this, concurrent deletion of NFATc3 and NFATc4 produced significant impairments in vascular development but also massive defects in sensory axon projection (49). NFAT signaling is recognized as an essential pathway both in the adult and developing nervous system. In the brain, NFAT-dependent gene regulation has been shown to play a critical role in neuronal survival, proliferation, and differentiation (54–56), as well as synaptogenesis, corticogenesis, and neurotransmission (57–59).

In human, the NFAT family encompasses five different members: NFAT1 [also called NFATc2 or NFATp), NFAT2 (also called NFATc1 or NFATc), NFAT3 (also called NFATc4), NFAT4 (also called NFATc3 or NFATx) and NFAT5 (also called tonicity enhancer binding protein (TonEBP) or osmotic response element-binding protein (OREBP)] that are encoded by separate genes. Moreover, the alternative splicing of each of these family members results in a different number of variants. There are eight possible variants of NFATc2 and NFATc1 in mammalian cells. The splicing of NFATc3 generates six isoforms, and up to twenty-four in the case of NFATc4 (60). On the other hand, sixteen various transcripts of NFAT5 have been identified so far (61) (**Table 1**). Specific NFAT isoforms appear to exhibit preferential associations with different types of neuronal cells (3, 10, 62, 63). For instance, NFATc3 is prominently expressed in astrocytes and pericytes, with comparatively lower expression in neurons. This isoform is also significantly upregulated in activated astrocytes (3). In contrast, NFATc4 has a broader distribution within neurons compared to NFATc3. NFATc1 and NFATc2, in particular, display a stronger preference for glial cells.

**Table 1:** General features of NFAT family members and their distribution in the brain.

NFAT member	Alternative name(s)	Number of variants	Regulated by	CNS distribution
**NFAT1**	NFATc2 and NFATp	8	Ca^2+^/Calcineurin	oflactory bulb, neuronal cell line
**NFAT2**	NFATc1 and NFATc	8	Ca^2+^/Calcineurin	hypothalamus, hippocampus, cerebellum, olfactory bulb, and frontal cortex
**NFAT3**	NFATc4	24	Ca^2+^/Calcineurin	olfactory bulb, cerebellum, and certain regions of the cortex
**NFAT4**	NFATc3 and NFATx	6	Ca^2+^/Calcineurin	hippocampus, retinal ganglion cells
**NFAT5**	TonEBP and OREBP	16	Osmotic stress	cerebral cortex, hippocampus, hypothalamus, substantia nigra, cerebellum, medulla oblongata

**Figure 2:**
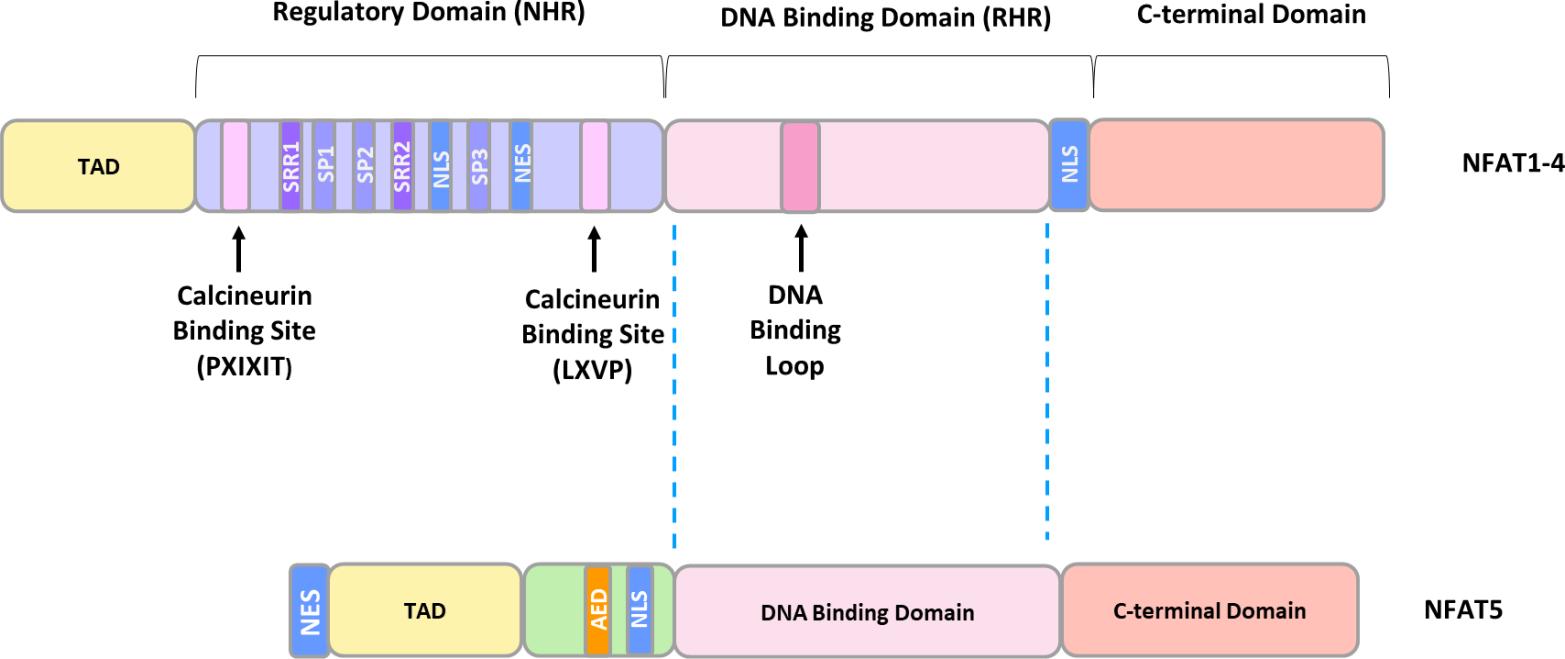
The general structure of NFAT proteins. TAD, transactivation domain; SRR, serine-rich region; SP, serine/proline motif; NLS, nuclear localization sequence; NES, nuclear exclusion sequence; AED, auxiliary export domain.

Despite having different C- and N-terminal sequences, all family members contain a highly conserved DNA-binding domain (DBD), similar to the one in the NFκB/Rel family, and are activated, with the exception of NFAT5, by CaN (47). NFAT5 lacks an essential CaN binding site, and its activity is regulated by changes in tonicity (64). Structurally, NFAT1-4 exist as monomers and possess two tandem domains: a regulatory domain located in the N-terminus, which is known as the NFAT homology region (NHR), and a Rel homology domain (RHR), where the DBD is located (65). The NHR contains two CaN binding motifs: a Ca^2+^-independent PXIXIT (where X denotes any amino acid) motif in the N-terminus, and a Ca^2+^-dependent LXVP motif in the C-terminal portion of the NHR (65). Furthermore, the NHR possesses the nuclear localization signal (NLS) responsible for CaN-dependent nuclear translocation, extended serine-rich regions (SRR1, SRR2), and three serine-proline motifs (SP1, SP2, SP3) (65) (**Figure 2**). NFAT dephosphorylation is believed to expose the NLS sequence, 265-KRK-267, which is located upstream of the SP3 motif. A second potential NLS site, 682-KRK-684 is situated near the C-terminus of NFATc1 (66). It is well-documented that phosphorylation status of the regulatory domain governs DNA-binding affinity and NLS exposure. However, the mechanism of NLS activation has not been elucidated at a structural level. The most plausible hypothesis suggests that dephosphorylation initiates global changes in NFAT conformation, facilitating its transition from an inactive to an active state (67). This transition may involve alterations in interactions within NFAT itself or with binding protein partners (68–70).

The NHR domain is moderately conserved and shares 22-32% sequence homology among the distinct NFAT members, whereas the Rel-homology region, consisting of approximately 270 amino acids, shares 64-72% sequence identity between various NFAT members (65, 71). In contrast, NFAT5 exists as a homodimer and possesses only a Rel DNA-binding domain structurally homologous to the Ca^2+^-dependent members, while the remaining 600 amino acids are entirely different (59).

Under resting conditions, the NHR domain is hyperphosphorylated within the SRR region and SP repeats, keeping the NFAT proteins in an inactive state and predominantly located in the cytoplasm (72). In response to elevated intracellular Ca^2+^, CaN becomes activated and dephosphorylates NFAT, leading to its rapid import into the nucleus (72). Once in the nucleus, NFATs can couple intracellular changes in Ca^2+^ concentration to the activation/repression of gene transcription, either acting alone or more frequently in cooperation with other transcription factors (73). Many of the NFAT transcriptional partners have not been characterized yet, but biochemical reconstructions have revealed several of them, including the cell life and death regulator activator protein 1 (AP1) and oncogenic regulators such as GATA binding protein 4 (GATA4) or myocyte enhancer factor 2 (MEF2) (46, 74). The nuclear accumulation of NFAT is counteracted by the synergistic actions of a set of protein kinases, including casein kinase 1 (CK1), glycogen synthase kinase 3 (GSK-3), and dual-specificity tyrosine-regulated kinases (DYRK) (55, 68, 75). These kinases can act in the cytosol to cause NFAT phosphorylation or in the nucleus to promote its rephosphorylation, facilitating nuclear export and transcription termination. CK1 functions as a maintenance and export kinase for NFATc2 (47). GSK-3 is classified as an export-type kinase, which phosphorylates the SP2 motif of NFATc2 and both, SP2 and SP3 motifs, of NFATc1 (47). In addition, phosphorylation of NFATc2 by GSK-3 requires previous phosphorylation by priming kinases including PKA or DYRK. Furthermore, DYRK1 functions as an export kinase which phosphorylates conserved SP3 motif in NFATc1 and NFATc2. In turn, DYRK2 can operate as a maintenance kinase, and phosphorylate the SP3 motif of NFATc1 and NFATc2 (47). Additionally, phosphorylation of SSR1 of NFATc1 by the c-Jun N-terminal kinase (JNK) or SSR1 of NFATc2 by the p38 mitogen-activated protein kinase (MAPK) has been shown to enhance cytosolic NFAT retention (69, 76, 77). However, it is not entirely clear whether kinases that phosphorylate NFAT under basal conditions also mediate its rephosphorylation in the nucleus and subsequent export back to the cytoplasm. Furthermore, NFAT transcriptional activity can be affected by different post-translational modifications, including acetylation or sumoylation, as well as phosphorylation events different from those regulating Ca^2+^-dependent translocation (78–80). It has been demonstrated that sumoylation plays a privileged role in extensive cellular processes and appears to be an important mediator of neuronal and synaptic function (81). In rat primary hippocampal neurons, sumoylation effectively suppressed the transcriptional activity of NFATc1, NFATc2, and NFATc3 isoforms, while in cortical neurons, only the transcriptional activity of NFATc1 and NFATc2 was affected by sumoylation (82). These findings indicate that the regulation of particular NFAT isoforms may be cell type-specific.

Regulation of subcellular localization and activity of Ca^2+^-dependent NFAT members is related to ligand binding to distinct cell-surface receptors (45, 46, 65, 83, 84). These receptors share a common feature: their ability to activate phosphatidylinositol-specific phospholipase C (PLC), thereby inducing Ca^2+^ entry through the plasma membrane. Activation of PLC triggers a cascade of events, including the hydrolysis of phosphatidylinositol 4,5-bisphosphate (PIP_2_) and the release of inositol 1,4,5-triphosphate (IP_3_), which in turn mobilizes intracellular Ca^2+^ through IP_3_ receptors located in the membrane of the endoplasmic reticulum (ER) (85). The increase in Ca^2+^ concentration leads to CaN activation by binding to its CaN-B regulatory subunit or by coupling Ca^2+^-dependent calmodulin to CaN. Efficient CaN-dependent dephosphorylation requires a docking interaction between NFAT and CaN. The PxIxIT sequence, located at the N-terminus of the NHR domain, serves as a primary docking site for CaN. Different NFAT members have unique PxIxIT sequences with a low affinity for calcineurin (Kd = 10–30 μM), which is essential to maintain sensitivity to environmental cues and restrict constitutive activation of NFAT (86). Aramburu and colleagues replaced the PxIxIT sequence of NFATc2 with VIVIT, a higher-affinity inhibitor identified through peptide selection. This peptide binds CaN with high affinity and efficiently competes with NFAT for CaN binding, thereby inhibiting NFAT nuclear accumulation (87). *In vivo* studies with transgenic mice carrying a mutation in the PxIxIT sequence of NFATc2 demonstrated enhanced cytokine production by differentiated T cells and exhibited deficiencies in embryonic and hematopoietic cell maturation (86). CaN dephosphorylates 13 out of 14 serines in NFATc2, which are conserved residues in all calcineurin-dependent NFAT members (67). It has been demonstrated that dephosphorylation of all 13 serine residues in NFATc2 is required to promote its nuclear localization (67). Furthermore, mutations of twelve serines to alanine, which imitate a nearly fully dephosphorylated state of NFATc2, lead to remarkable nuclear accumulation in both, resting and CsA-treated cells.

It is not fully understood whether site-specific dephosphorylation is an organized mechanism and what the consequences are for downstream signaling in specific cell types. However, the experiments using mass spectrometric analysis indicate that the SRR1 region, which is closely adjacent to the main CaN docking site, is preferentially dephosphorylated at low CaN activity (67). NFAT mutants with restricted deletions or S → A mutations in the SRR1 motif are more susceptible to dephosphorylation in the SP repeats by CaN compared to wild-type NFATs (87, 88). CaN-regulated NFAT activation is particularly important in resting conditions when the rise in Ca^2+^ due to its release from intracellular stores is not sufficient for direct NFAT activation (89).

**Figure 3:**
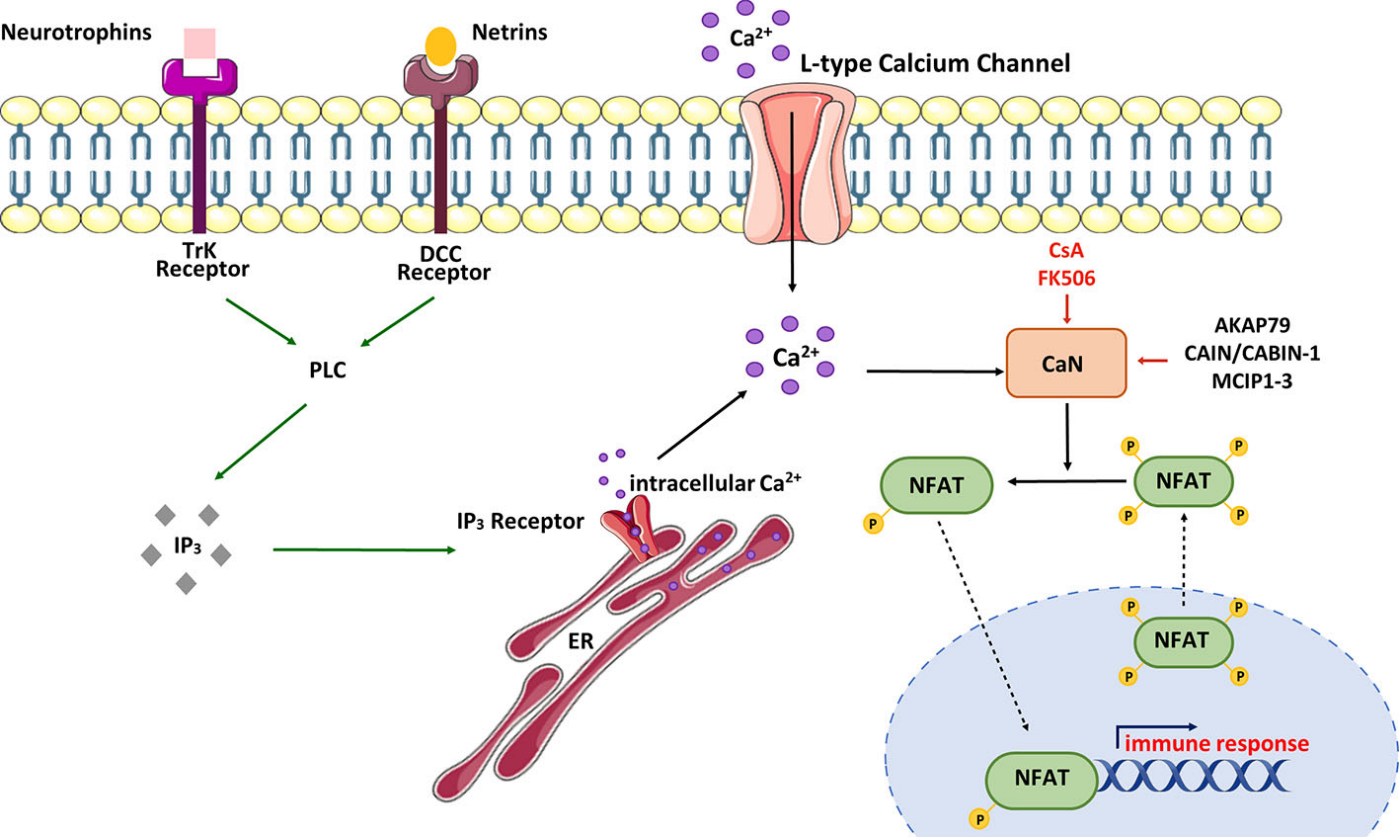
Schematic view of CaN/NFAT activation cycle. NFAT is activated in response to cell-surface receptors coupled to intracellular Ca^2+^ mobilization. Activated phospholipase C (PLC) generates inositol-1,4,5-triphosphate (IP_3_), which binds IP_3_ receptors located in the endoplasmic reticulum (ER). Stimulation of IP_3_ receptors produces a brief spike in Ca^2+^ by depleting the ER stores. Ca^2+^ increases promote activation of calmodulin-dependent enzymes including calcineurin (CaN). CaN dephosphorylates multiple serines in NFAT protein leading to its nuclear import and activation of NFAT-dependent transcription of genes involved in immune response. NFAT rephosphorylation by multiple kinases triggers its nuclear export and cytosolic retention. CaN activity can be modulated by immunosuppressive drugs – cyclosporine A (CsA) and tacrolimus (FK506) or CaN-interacting proteins – AKAP79 (A-kinase anchoring protein 79), MCIPs (modulatory calcineurin-interacting proteins) or CAIN/CABIN-1 (endogenous inhibitor of CaN activity). In neurons, NFAT can be selectively activated by Ca^2+^ influx through L-type Ca^2+^ channels.

The parameters for Ca^2+^/CaN/NFAT activation can be regulated in various ways (**Figure 3**).

In neurons, NFAT activation occurs as a result of stimulation of tyrosine kinase receptors (Trk) coupled to PLCγ (90). Neurotrophins and netrins are upstream components of this signaling pathway. For example, it has been observed that overexpression of the TrkA receptor in cortical neurons supports NFAT transcriptional activation upon nerve growth factor (NGF) treatment (91). Additionally, Groth et al. (92) demonstrated that treatment of cultured spinal neurons with NGF enhanced NFAT-dependent transcription. Brain-derived neurotrophic factor (BDNF) has also been found to increase NFAT transcriptional activity in cultured cortical neurons, and this activation was abolished by co-treatment with CaN inhibitors such as CsA or FK506 (91). To illustrate the neurotrophic significance in the NFAT signaling pathway, Graef and colleagues (91) utilized an EGFP-NFATc4 hybrid to visualize NFATc4 subcellular localization in responsive cortical neurons. The data showed that NFATc4 was imported into the nucleus, and its transcriptional activity was enhanced in response to BDNF stimulation. Moreover, cultured hippocampal pyramidal neurons treated with BDNF also exhibited increased endogenous transcriptional activity of NFATc4 (92). It is worth mentioning that inhibition of PLC activity or depletion of intracellular Ca^2+^ stores attenuated the ability of BDNF to activate endogenous NFAT in hippocampal neurons (92, 93). These results collectively demonstrate that NFAT transcriptional complexes appear to be downstream of neurotrophin signaling, and their activity in neuronal development is regulated through PLC-dependent mechanisms.

A growing body of evidence indicates that NFAT transcription factors can also play a pivotal role in integrating signaling pathways involved in neuronal growth driven by guidance cues during synaptogenesis. In this process, molecules such as netrins are crucial for axon guidance and control during the development of neuronal circuits (94). Supporting this, failure in the elongation of commissural axons caused by triple deletion of NFAT (c2/c3/c4) was seen in mice bearing mutation in netrin-1 or netrin DCC receptor (95, 96). Additionally, it has been demonstrated that netrin-dependent axon extension in E13 rat dorsal spinal cord explants required CaN/NFAT signaling, and stimulation with netrin-1 promoted NFAT transcriptional activity via the DCC receptor (91).

The emptying of ER Ca^2+^ stores is a known trigger that initiates the process of capacitative Ca^2+^ influx across the plasma membrane, referred to as store-operated Ca^2+^ entry. In neurons, Ca^2+^ influx may also occur via voltage- or ligand-gated Ca^2+^ channels, each of which is regulated in a precisely coordinated manner. It is well recognized that neuronal L-type voltage-gated Ca^2+^ channels (LTCC) play a crucial role in various synaptic processes underlying learning and spatial memory formation in the hippocampus and other brain areas (97, 98). The activation of LTCC by high extracellular K^+^ triggers the nuclear localization of NFAT in cultured hippocampal and DRG neurons (93, 99, 100). It has also been demonstrated that *N*-methyl-D-aspartate receptor stimulation leads to enhanced nuclear import of NFATc3 and NFATc4 in cortical neurons (101).NFAT activity can also be affected by intracellular inhibitors of CaN. Among the numerous endogenous proteins that have a pivotal role in regulating CaN activity is A-kinase anchoring protein 79 (AKAP79), which binds CaN and blocks its access to the substrates (102). It is worth mentioning that AKAP79 also binds protein kinase A (PKA), which is an NFAT kinase that prevents its nuclear import (103). Other regulators, such as CAIN/CABIN-1, can potentially inhibit CaN activity in a phosphorylation-dependent manner (104) and exert a similar effect on NFAT dephosphorylation and translocation, like the regulators of calcineurin (RCANs) or modulatory calcineurin-interacting proteins (MCIPs; MCIP1–3) (59). Studies have shown that RCANs may either positively or negatively affect the activation of CaN/NFAT signaling. Although the mechanistic explanation of how these proteins modulate CaN activity is still questionable, RCANs were found to participate in CaN biosynthesis or recycling, playing a role as chaperones (59).

## Pharmacological inhibitors of CaN/NFAT with potential use in AD treatment

Relatively insensitive to classical inhibitors of phosphatases, CaN activity can be corroborated by two widely used immunosuppressive drugs: cyclosporine A (CsA) and FK506 (tacrolimus) (105, 106). CaN/NFAT signaling may also be inhibited by other molecules that differ in chemical properties and the mechanism of action (**Table 2**).

**Table 2:** Physicochemical characteristics of CaN/NFAT inhibitors.

Inhibitor	Characteristic profile				
	Composition	Mode of action	Binding partner	IC_50_/K_d_^*^	Side effects
**CsA**	A cyclic peptide of 11 amino acids	Complex blocks substrate access to the active centre of CaN	Cyclophilin	7 nM	Nephrotoxicity, hypertension, neurotoxicity
**FK506**	23-membered polyketide macrolide	Complex blocks substrate access to the active centre of CaN	FK506 binding protein (FKBP-12)	0.4 nM	Nephrotoxicity
**VIVIT peptide**	A 16 member linear L-peptide	Blocks CaN-NFATc interaction	No	0.5 µM* < 1 µM	Not observed
**LxVP peptide**	15 - mer peptide from NFATc1	Blocks CaN-NFATc interaction and regulates enzymatic activity of CaN	No	~ 0.3 µM	Not observed
**Dipyridamole**	Pyramidopyri-midine compound	Disrupts CaN-NFATc binding	No	~ 10 µM	Cytotoxicity, excitotoxicity under injurious conditions
**INCA-6**	Small organic molecule	Disrupts CaN-NFATc complex formation by covalent binding to CaN	No	~ 0.8 µM*	Cytotoxicity
**A-28522**	Small organic molecule	Inhibits NFAT activity	No	N/A	N/A
**Q-134R**	Hydroxyquinoline derivative	Partially inhbits NFAT activity	No	~ 400 nM	Not observed

N/A, not available.

*Kd.

### Cyclosporine A

CsA has been employed in the treatment of organ transplant rejection since 1976 when Borel and colleagues demonstrated its immunosuppressive properties (107). The mechanism of action of CsA depends the formation of a complex with cytosolic proteins known as cyclophilins, particularly the 17 kDA cyclophilin A, which is highly abundant in T cells (108, 109). Cyclophilins, classified as immunophilins, possess peptidyl-proline-*cis*-*trans* isomerase (PPIase) activity that plays a role in ensuring proper protein folding (110). It has been established that CsA inhibits PPIase, although this effect is not associated with the mechanism of immunosuppression. The formation of a ternary complex involving CsA, cyclophilin A and CaNA inhibits calcineurin phosphatase activity and blocks the transcription of cytokine genes, including IL-2 and IL-4 (111). In addition to its impact on the CaN/NFAT pathway, CsA affects the activity of NF-κB and AP-1, transcription factors essential for the regulation of IL-2 gene expression (112, 113). CsA has been demonstrated to inhibit JNK and p38 MAPK signaling, as well as an antigen-specific Ca^2+^-independent response (114, 115). Several early reports have indicated both pro-apoptotic and neuroprotective effects of CsA in neuronal and neuronal-glia mixed cultures (116–121). The pharmacological normalization of CaN activity can offer a certain degree of neuroprotection (122, 123), improve synaptic function (124), and reduce glial activation (125, 126) in experimental models of brain degeneration. However, despite the undeniable benefits of CsA, its use as an immunosuppressant is limited by potential side effects, including nephrotoxicity, neurotoxicity, and hepatotoxicity (127).

### FK506 (tacrolimus)

Structurally, FK506 is a macrolide antibiotic with immunosuppressive properties. It forms a complex with immunophilin FK506 binding protein (FKBP12), and the FK506-FKB12 complex suppresses CaN-dependent NFAT signaling. Despite significant differences in structure and mechanism of action, CsA and FK506 surprisingly share similar biological effects (128, 129). However, FK506-induced immunosuppression is not solely due to the inhibition of the CaN/NFAT pathway but also involves other T-cell activation pathways (130). Unlike CsA, FK506 inhibits IL-2 induced IL-5 production by human CD4^+^ T cells and T-cell proliferation stimulated by IL-2 and IL-7 (130). Moreover, FK506, but not CsA, decreases the expression of human-affinity IL-7 receptor and is more effective in controlling IL-2 production by T cells in patients receiving kidney transplantation. These data suggest that at least some of FK506’s effects are mediated by CsA-insensitive signaling pathways (131). Regarding the immunosuppressive effects, it is evident that FK506 hinders processes further downstream in the T-cell activation cascade, beyond CaN activation. This is supported by the observed capacity of FK506 monotherapy to effectively manage steroid-resistant allograft rejection episodes, which is a critical distinction from CsA, as CsA lacks efficacy in the treatment of allograph rejection (132).

### VIVIT and LxVP peptides

The VIVIT is a 16-mer oligopeptide (MAGPHPVIVITGPHEE) developed through an affinity- driven selection of peptides encoding mutated versions of the wild NFAT protein sequence (SPRIEIT), which is characterized by a low affinity toward the PxIxIT consensus motif (87, 133). VIVIT exhibits a remarkable 25-fold higher potency in inhibiting NFATc dephosphorylation compared to the original SPRIEIT peptide. While 10 μM CsA/cyclophilin can completely inhibit CaN activity, it remained unaffected even at a concentration of up to 100 μM VIVIT. However, the presence of 100 μM VIVIT is sufficient to disrupt the interaction between CaN and NFAT, thus preventing NFAT dephosphorylation (87). Furthermore, VIVIT demonstrates superior selectivity in inhibiting NFAT compared to CsA and FK506. VIVIT has been demonstrated to inhibit the dephosphorylation of NFATc1, NFATc2 and NFATc3, as well as the nuclear import of NFATc2. Additionally, GFP-VIVIT suppresses NFAT-mediated expression of IL-2, IL-3, IL-13, TNF-α, macrophage inflammation protein 1 (MIP-1 α), and granulocyte-macrophage colony-stimulating factor (GM-CSF) in Jurkat T cells while not affecting CsA-regulated genes such as TNF-β and lymphotoxin-β (87). To date, there have been no reported toxic side effects associated with VIVIT. Its outstanding specificity in disrupting NFAT signaling without CaN activity in a general sense, makes VIVIT highly promising for reduced toxicity compared to CsA or FK506. Similarly, its cell-permeable analog, 11-R VIVIT, has demonstrated a lack of toxicity at doses up to 10 mg/kg (134). In this analog, VIVIT was fused to the C-terminal of a poly-arginine-based protein transduction domain (11R-VIVIT, RRRRRRRRRRR-GGG-MAGPHPVIVITGPHEE).

LxVP peptides are derived from CaN-docking NFATc sequence LxVP, and they compete for binding with activated CaN (135, 136). Notably, CaN exhibits a strong affinity for the LxVP motif of NFATc1, NFATc3 and NFATc4, while its binding affinity is considerably weaker for NFATc2, the predominant isoform in activated CD4^+^ T cells (137). For instance, the GST-LxVPc1 peptide, consisting of 15 amino acids from human NFATc1, shows more efficient binding to CaN compared to any of the PxIxIT motifs found in NFATc1-c4. In contrast, the GST-LxVPc2 fusion peptide from NFATc2 does not bind to CaN under the same conditions. The LxVP effectively inhibits CaN activity when assessed with a phosphopeptide derived from the PKA regulatory subunit II, containing phosphoSer-95, and it increases phosphatase activity when evaluated using a nitrophenyl phosphate assay (135, 136). Therefore, similar to CsA and FK506, LxVPc1 can modify CaN’s activity through an interaction with a site distinct from the active site. When GFP-LxVPc1 fusion protein is overexpressed in HeLa cells, it effectively inhibits NFATc2 dephosphorylation and nuclear translocation upon ionophore treatment. Similarly, in Jurkat T cells, it inhibits NFATc2 dephosphorylation and the expression of luciferase under the control of the IL-2 upon PMA/ionophore stimulation (135, 136).

### Small molecular inhibitors acting on CaN molecule

Dipyridamole is a medication that serves as an inhibitor of nucleoside transport and a PDE3 (phosphodiesterase 3) inhibitor. When administered chronically, it effectively prevents the formation of blood clots, and when given in high doses over a short period, it induces the dilation of blood vessels. When combined with aspirin, it is an FDA-approved treatment for secondary prevention of stroke (138). In a screening of the Prestwick Chemical Library, which contains 880 biologically active molecules, Mulero and colleagues identified dipyridamole as a compound that competes with CaNA for binding to the RCAN1 peptide (139). Functional assessments of its inhibitory effect on CaN activity revealed that dipyridamole does not interferes with phosphatase activity. Its mechanism of action appears to involve a specific interaction with NFAT substrates. In line with this, dipyridamole inhibits NFAT nuclear translocation, NFAT activity and the expression of NFAT-dependent cytokine genes in human T cells (139). It has been suggested that, beyond its antiplatelet activity, this drug may also have an immunomodulatory effect *in vivo* (140, 141).

### INCA

The **I**nhibitors of **N**FAT-**C**alcineurin **A**ssociation (INCAs) have been selected based on their ability to compete with the VIVIT peptide for binding to CaN. Among these, three compounds, namely INCA-1, INCA-2 and INCA-6 are capable of completely displacing VIVIT from CaN, and they achieve this at low micromolar concentrations by inducing an allosteric change in the NFAT-binding site (142). It is important to note, that the binding site of INCA1,2 and 6 is centered on Cys-266 of CaNA and does not involve PxIxIT core motif. Interestingly, particular INCAs differ in their mechanisms of action. For instance, INCA-6 is known to act on NFAT activity whereas the physiological effects of INCA2 are associated with a general inhibition of CaN activity (142). However, concentration-dependent cytotoxicity has been reported for all INCAs, suggesting potential limitation in their use in biological systems (142, 143).

### Small molecular inhibitors not acting directly on CaN

A-285222, a member of bis-trifluoromethyl-pyrazole class, falls within the category of immunosuppressive agents known for their ability to inhibit NFAT activity in both human and non-human primate cells through a calcineurin-independent mechanism (144). When applied to intact T cells, A-285222 maintains NFAT in a phosphorylated state within the cytosol, preventing its nuclear accumulation, all while leaving AP-1 or NF-κB activation unaffected. Consequently, this drug effectively blocks NFAT-dependent cytokine gene transcription. It is important to note that while the drug shows potential as an immunosuppressive agent, it is associated with severe side effects, particularly neurotoxicity. Nevertheless, various cell-specific effects have been demonstrated (145–147).

Q134R is a hydroxyquinoline derivative known for its ability to penetrate the brain and inhibit both the induction of NFAT transcriptional activity and caspase-3 activity (148). Importantly, this drug does not have any impact on CaN itself, which means it avoids the immunosuppressive adverse effects associated with other agents. Preclinical studies have showed its safety, good stability, and acceptable oral bioavailability. Phase I/A trials have been successfully completed, and the drug is currently progressing into phase II trials.

## Contribution of NFAT isoforms to neuronal death in AD

Accumulating evidence suggests that NFAT activity is involved in several neuropathologies (59). In nervous tissue, abnormalities in CaN/NFAT signaling are increasingly related to a variety of pathological features associated with Alzheimer’s disease (AD), including synaptic dysfunction, glial activation, and cell death (11) The prevalence of studies, as will be discussed in subsequent sections, indicates a causative relationship between NFAT and AD, and some of the first evidence has been provided by Abdul and colleagues who demonstrated a relationship between amyloid toxicity and NFAT signaling (10).

A postmortem study of human hippocampal tissue revealed increased nuclear translocation of individual NFAT members at different stages of AD, which correlated with the severity of cognitive decline (11). NFATc2 activation is especially critical in the early stages of AD, while selective NFATc4 activation occurs later when the control of neuronal Ca^2+^ homeostasis and reactive oxidative species production is lost, leading to further neurodegeneration, neuronal death, and ultimately, dementia (11). Moreover, levels of several cytokines increase as NFATc2 accumulates in the nucleus (11), which is consistent with its role in neuroinflammation occurring during the early stage of AD. Excessive activation of NFATc2/c4 at different stages of AD progression may contribute to synaptic dysfunction and cognitive decline.

In agreement with this hypothesis, inhibition of CaN/NFAT signaling pathway at each stage of AD progression should prevent degeneration of neuronal processes and slow down cognitive decline. Numerous studies on animal models of AD have demonstrated that CaN inhibitors, such as tacrolimus, showed neuroprotective and/or cognitive enhancing properties and extended lifespan (123, 149–151). In addition, Abdul et al. suggested that Aβ-induced neurodegeneration is associated with selective changes in NFAT signaling (10). In line with that, over-activation of CaN, enhancing nuclear localization of NFATc2 and NFATc4, correlated with increased dementia severity in the human hippocampus, while the subcellular localization of NFATc1 was cytoplasmic (10). Increased NFATc2 activation was revealed in AD patients with mild cognitive impairments, whereas NFATc4 showed a high nuclear accumulation in patients with severe dementia and AD (10).

While these results indicate the differential contribution of NFAT isoforms to AD pathology, other reports suggest that NFAT may be an essential component of signaling pathways pharmacologically targeted in AD treatment. Lithium has been proposed as a treatment for AD and other neurodegenerative disorders (152). However, its clinical use is limited due to a high rate of associated adverse side effects, and the mechanisms underlying those effects are not fully understood. It has been demonstrated that lithium can act as a repressor of GSK-3, with K_i_ values for GSK-3α (~3.5 mM) and GSK-3β (~2.0 mM) that exceed therapeutic lithium serum concentrations in humans (0.5–1.2 mM) (153–155). However, lithium, like other small molecule inhibitors, is unable to selectively suppress the activity of GSK-3 isoforms, and its impact on cognitive function is lost in aged AD transgenic mice (156). For these reasons, genetic approaches have been employed to assess whether there are isoform-specific effects of GSK-3. While the majority of these studies focuses on the contribution of GSK-3β, a growing body of evidence indicates a distinct role for both isoforms in Aβ pathology (157–160). For instance, *in vitro* and *in vivo* studies have revealed that hyperphosphorylation of tau promoted by GSK-3β leads to the formation of neurofibrillary tangles, which eventually trigger neurodegenerative conditions (161–167). It has also been reported that cleavage of the amyloid precursor protein (APP) into beta-amyloid peptide is meditated by GSK-3 β (166). In addition to APP, presenilin-1 (PS1) is involved in the aggregation of the pathogenic Aβ peptide and is regulated by GSK-3β (168, 169). This is supported by the study of Ly and colleagues, which showed that specific suppression of GSK-3β, but not GSK-3α, reduced Aβ formation via NF-αB-dependent transcriptional control of β-secretase 1 (170). In contrast, Hurtado et al. (171), using AAV-delivered shRNAs and GSK-3α conditional knockout mice, suggested that GSK-3α plays a predominant role in AD pathology. However, this study was limited by the use of newborn AD model mice and putative compensatory changes in the other GSK isoform. Given that overactivated GSK-3 is hypothesized to play a central role in the pathogenesis of AD (159), the beneficial effect of lithium would stem from the normalization of GSK-3 activity and the prevention of progressive Aβ production as well as local microglial-mediated inflammatory responses.

Transgenic mice with conditional expression of dominant-negative GSK-3β showed increased neuronal apoptosis and impaired motor coordination, a phenotype that can be reversed by the expression of a dominant-negative GSK-3β form (172). It has also been hypothesized that treatment with therapeutic levels of lithium can promote neuronal loss through GSK-3β inhibition (152). These observations strongly indicate that GSK-3 activity is critical for the viability of adult neurons, and any manipulations beyond physiological GSK-3 levels may pose a potential risk of neurological toxicity. A similar effect was observed in different cell types, including Jurkat cells, differentiated immortalized hippocampal neurons (173), HL-60 promyeoloblast cells (174), human lung carcinoma A549 cells (175), human choroidal melanoma cells (176), human leukemia NB4 cells (177), K562 human erythroleukemia cells (178), human cardiomyocytes (179) and multiple myeloma (180). On the other hand, an expanding body of literature has provided substantial evidence that lithium can confer neuroprotection against various insults [for a comprehensive review, refer to (181)].

Interestingly, lithium-induced apoptosis and motor deficits were associated with elevated nuclear accumulation of NFATc4 and NFATc3, leading to increased Fas ligand (FasL) expression and its activation (152). The opposite effect was observed in mice lacking the Fas-receptor (*lpr* mice) or following CsA treatment. In addition, activation of the CaN/NFAT/FasL death signaling has been suggested in methamphetamine-induced neuronal apoptosis (182). The link between FasL regulation by NFAT is interesting as Fas and FasL are associated with neuronal degeneration in the AD brain and participate in Aβ-mediated cell death (183). *In vivo* treatment with CaN or NFAT inhibitors abolished NFAT-mediated FasL expression and attenuated neuronal apoptosis (184).

Experimental evidence suggests a possible role of NFATs in mediating the survival response. It has been demonstrated that in the presence of growth factors and neuronal activity mimicked by high extracellular KCl concentrations, both endogenous NFATc4 and exogenously expressed NFATc4 were localized to the nucleus of granule neurons (54). On the contrary, serum/K^+^ deprivation led to NFATc4 nuclear export, which was strongly associated with the induction of neuronal apoptosis. Treatment with the GSK-3 inhibitor lithium chloride blocked the nuclear export of NFATc4 and cell death, suggesting a correlation between NFAT localization and neuronal survival. In line with that, NFATc4 knockdown resulted in enhanced apoptosis, observed even in a rich culture medium and high K^+^. The expression of a constitutively active form of NFAT prevented neuronal cells from apoptosis induced by low K^+^ or growth factor deprivation (54).

## CaN/NFAT signaling and Aβ neuropathology

CaN/NFAT signaling has been recognized as a target for Aβ pathology in numerous studies [for review, see (3, 11)], highlighting the importance of Aβ-mediated Ca^2+^ homeostasis deregulation, which creates a favorable environment for CaN/NFAT activation. A report by Wu and colleagues showed that short exposure of primary cortical neurons to Aβ oligomers led to the dynamic progression of CaN activation and resulted in morphological changes in spines and postsynaptic proteins, while longer exposure led to NFAT accumulation in the nucleus and significant spine loss (35). Similarly, the loss of spines and dendritic branching simplification evoked by Aβ treatment in primary neurons were mimicked by constitutively active NFAT (185). Transfection with VIVIT-GFP before Aβ exposure greatly improved neuronal morphology despite the persistence of Aβ in the culture medium. Spine atrophy and neuronal abnormalities seen in the vicinity of amyloid plaques in APP/PS1 mice were prevented when AAV2-VIVIT was delivered by stereotactic intracortical injections (185). This suggests that selective disruption of CaN-NFAT interaction may be protective against Aβ synaptotoxicity. These findings also illuminate a possible mechanism underlying the lack of cognitive improvement by GSK-3 inhibitors in an AD clinical trial (186). Notably, Aβ-activated GSK-3 potentiates further Aβ production, creating a positive feedback loop. Activated GSK-3 phosphorylates NFAT, inhibiting CAN/NFAT apoptotic pathway, and consequently, GSK-3 inhibitors release this inhibitory brake, promoting NFAT-mediated neurodegeneration (186). Similarly, Aβ treatment of murine hippocampal neurons caused dysregulation in intracellular Ca^2+^ and impaired dendritic and axonal transport of BDNF (187). Exposure of mixed neuron/astrocyte culture to Aβ stimulated CaN/NFAT activity and triggered CaN proteolytic cleavage, resulting in an overall increase in intracellular CaN activity (188). The accumulation of both CaN and NFATc4 (but not NFATc2) in the nucleus of hippocampal tissue positively correlated with a higher level of soluble Aβ, which steadily increased as the severity of dementia progressed (10).

**Figure 4:**
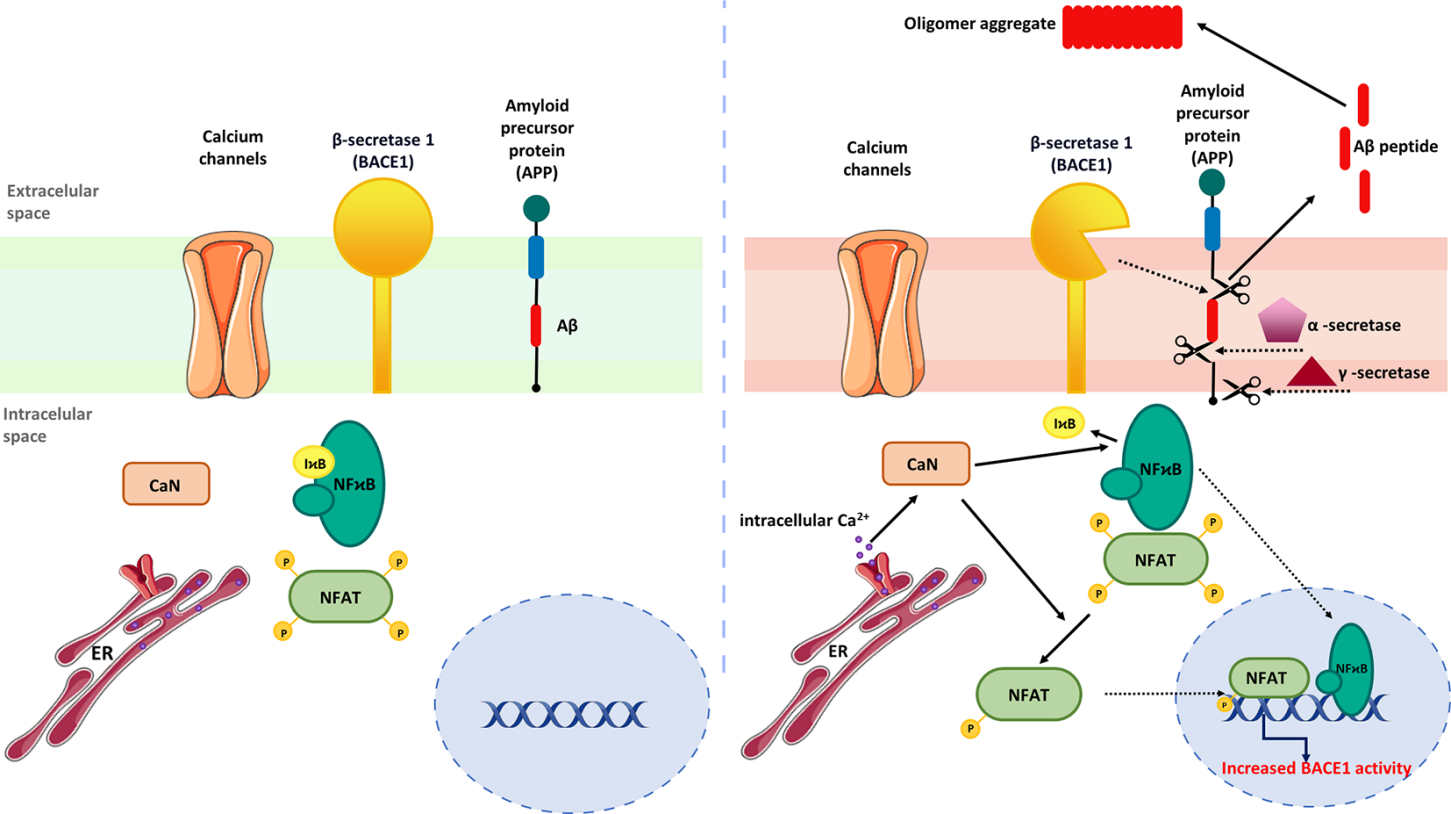
Regulation of astrocytic Aβ metabolism by CaN/NFAT signaling. (Left) In steady-state conditions, BACE expression is maintained at low level. (Right) Activation of astrocytes by upstream inflammatory mediators stimulates NFAT and NF-κB nuclear translocation and BACE1 upregulation. Increased BACE1 activity is associated with abnormal production of Aβ, which subsequently form amyloid plaques.

Mounting evidence indicates that activation of CaN/NFAT signaling may be linked to enhanced generation of Aβ peptides (3, 36). To support this, Hong and colleagues (189) showed that FK506 administered to 8-month-old APP/PS1 double transgenic mice reduced Aβ accumulation in the cortex and hippocampus while increasing matrix metalloproteinase-9 (MMP-9) expression, which is known to degrade Aβ (190). Moreover, increased MMP-9 colocalized with astrocytic marker GFAP (glial fibrillary acidic protein) in FK506-treated mice indicating that drug-dependent MMP-9 up-regulation originates from activated astrocytes. In line with that, no correlation between MMP-9 immunoreactivity and the neuronal marker NeuN was observed, though FK506 increased the level of PSD-95 and synaptophysin, proteins involved in postsynaptic density formation and vesicular neurotransmitter release, respectively. Similarly, intrahippocampal injections of AAV2-VIVIT under the astrocyte-specific promoter reduced Aβ pathology, normalized spontaneous synaptic activity, prevented dendritic degeneration, and improved cognition in the 5xFAD mouse model of AD (36). Furthermore, AAV2-mediated NFAT inhibition increased glutamate transporter-1 expression in hippocampal astrocytes and reduced the number of glutamate-evoked transients (36), suggesting CaN/NFAT-dependent loss of glutamate regulatory properties underlying hyperexcitability in AD. Another study pointed out astrocytic CaN/NFATc4 signaling activated in response to Ca^2+^ overload as one of the key pathological mechanisms driving Aβ generation by inducing β-secretase 1 (BACE1) transcription (191) (**Figure 4**).

BACE1 protein and activity were found to be elevated in the AD brain, suggesting that BCAE1 up-regulation may be a phenomenon occurring early in AD or accelerating AD progression (192). Importantly, BACE1^-/-^ mice are devoid of Aβ amyloidosis, electrophysiological dysfunctions, and cognitive deficits (192), implying targeted BACE1 inhibition to improve Aβ-mediated loss of cognitive function in humans with AD. Although not verified in AD models, Mei and coworkers demonstrated that NFATc4 directly binds the BACE1 gene promoter and regulates its expression (191). It is worth noting that the development of potent BACE1 inhibitors presents numerous challenges. One of the major hurdles is the structural similarity of BACE1 to other aspartyl proteases, a family that includes several enzymes widely expressed through the human body, such as BACE2, pepsin, renin, cathepsin D, and cathepsin E (193). Thus, it is imperative to develop effective BACE1 inhibitors without affecting other proteases and to exclude off‐target side effects (194, 195). Furthermore, another aspect of discovering successful BACE1 inhibitors is the relativity large size of the BACE1 active site (195). Moreover, another limiting factor is related to the ability of these molecules to pass through the blood-brain barrier (196). To date, despite tremendous efforts, none of the BACE1 inhibitors has succeeded in demonstrating clinical value.

## CaN/NFAT in AD-associated inflammatory signaling

Many studies now point to the contribution of neuroinflammation to the progression of neuropathological changes observed in AD. One of the signs that emerges early in the course of AD is astrocyte activation, which becomes more prominent during later stages when the amyloid and tau pathology is extensive (197). It is hypothesized that CaN/NFAT signaling serves as a critical mechanism triggering astrocyte phenotype switching and plays a pivotal role in astrogliosis and brain neuroinflammation. In astrocytes and microglia, CaN/NFAT is activated in response to a variety of neuroinflammatory mediators, including pro-inflammatory cytokines, glutamate, ATP, S100 protein, thrombin, vascular-injury factors, and Aβ. Once activated, CaN/NFAT may further potentiate the inflammatory response by driving the expression of numerous inflammatory factors, many of which are elevated in AD [reviewed in (198)]. It is well-accepted that Ca^2+^ homeostatic imbalance may aggravate AD through the pathological activation of neuronal networks but, in contrast to neurons, little is known about Ca^2+^ signaling specifically linked to glial CaN/NFAT activation. L-type voltage-dependent Ca^2+^ channels are likely to play a role, but the contribution of other plasma membrane transporters as well as those located in the ER cannot be ruled out (199).

**Figure 5:**
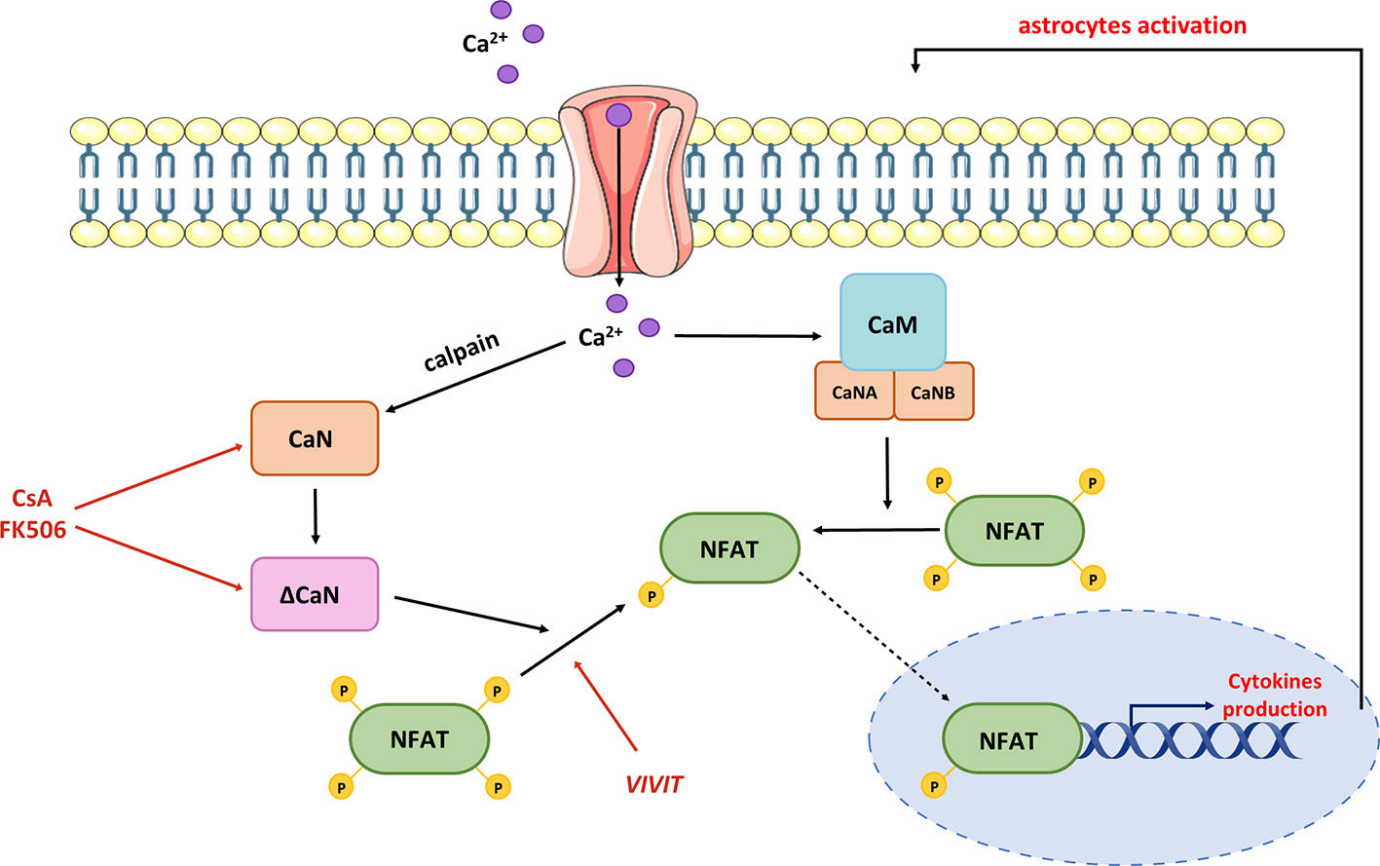
Contribution of hyperactive CaN/NFAT to pro-inflammatory cytokine production. Dysregulation of Ca^2+^ homeostasis leads to the production of truncated CaN fragment (ΔCaN), which is constitutively active. Hyperactivated CaN/NFAT sustains prolonged astrocytic activation and chronic neuroinflammation through a continuous stimulation of pro-inflammatory cytokine genes. Normalization of CaN/NFAT activity may be provided by commercial immunosuppressants such as cyclosporine A and tacrolimus (FK506), or the VIVIT peptide.

Furthermore, CaN-dependent signaling may be a nodal point linking Ca^2+^ dyshomeostasis, Aβ accumulation, and neuroinflammation. In primary neurons, Aβ treatment stimulated a Ca^2+^-dependent protease calpain, which cleaves C-terminal autoinhibitory domain, producing a constitutively active truncated version of CaN (ΔCaN, 48 kDa CN-Aα) insensitive to Ca^2+^/CaM regulation (200) (**Figure 5**).

High levels of ΔCaN were detected in areas surrounding amyloid plaques in mouse and human brains (201, 202). Moreover, the levels of ΔCaN and active calpain correlated with one another in human hippocampal tissues of individuals with mild cognitive impairments when compared to age-matched controls (188). These changes were further linked to increased proteolysis of the NMDA receptor subunit 2B, which is necessary for long-term potentiation and memory formation (203, 204). This provides a mechanism by which oligomeric Aβ may contribute to brain degeneration through calpain-mediated CaN proteolysis and further overactivation of CaN/NFAT signaling. However, the molecular phenotype of ΔCaN-expressing astrocytes and their role in AD pathology have not been thoroughly studied. In primary neuronal cultures, heterologous expression of ΔCaN activated the transcription of several genes involved in immune response and morphogenesis (205). As many of these transcripts encode releasable cytokines and chemokines, CaN/NFAT signaling in activated astrocytes may contribute to cerebral vascular damage, reducing micronutrient delivery and compromising neuronal-glia interaction. The hypothesis of impaired microcirculation underlying AD development was postulated nearly 30 years ago (206) and has been widely discussed in multiple studies (207, 208). The plausible role of CaN/NFAT in AD-related vascular pathologies is further supported by recent work showing the upregulation of ΔCaN and NFAT signaling in astrocytes nearby regions of small vessel damage (209). Further evidence of aberrant astrocytic Ca^2+^/CaN/NFAT signaling contributing to vascular pathology associated with cognitive decline and dementia comes from the latest study of Sompol and colleagues (210). Using a diet-based model of hyperhomocysteinemia (HHcy), which recapitulates numerous features of AD, they demonstrated that VIVIT-mediated NFAT inhibition ameliorated astrocytic reactivity and improved blood flow in arterioles and capillaries. Moreover, the suppression of NFAT signaling preserved CA1 synaptic function and improved the cognitive performance of HHcy diet mice.

In addition to CaN overactivation studies, inhibition with either pharmacological drugs or targeted peptides revealed a similar relationship between CaN/NFAT and neuroinflammation. Targeting AAV2-VIVIT peptide to hippocampal astrocytes in APP/PS1 mice reduced glial activation, lowered Aβ levels, and improved cognitive and synaptic function (211). These findings align with previous reports showing high levels of CaN in activated astrocytes and its role in phenotype switching via NFAT signaling (46, 84, 205). Although the upstream signals leading to pathological activation of CaN in astrocytes are not well-characterized, heterologous expression of a constitutively active form of CaN upregulated genes linked to the activated phenotype, as well as numerous inflammatory-related genes (3). Astrocytic CaN/NFAT signaling has also been demonstrated to mediate the neurotoxic effects of several factors implicated in AD pathogenesis, including TNFα, CCL2, Cox2, GM-CSF, IL-6, IL-1β, and other cytokines (62, 202, 212–214). For instance, IL-1β promoted CaN/NFAT activation in primary astrocytes through IL-1 receptors and L-type Ca^2+^ channels without affecting CaN or NFAT expression levels (212). Interestingly, activated CaN/NFAT caused robust activation of CaN/NFAT in adjacent astrocytes via paracrine signaling. This observation places CaN/NFAT in the center of a positive feedback loop controlling local production of neuroinflammatory mediators.

An interesting approach based on reprogramming macrophages to become anti-inflammatory was proposed in a study conducted by Escolano and coworkers (215). By introducing the LxVP peptide that interferes with CaN-NFAT binding (for exact mechanism of action see *Pharmacological inhibitors of CaN/NFAT with potential use in AD treatment* section) they demonstrated that systemic or local peptide delivery via adenoviral gene transfer attenuated the inflammatory response *in vivo*. Mechanistically, LxVP-mediated CaN inhibition reduces the activity of MKP-1, a dual specificity protein phosphatase, releasing p38 MAPK kinase from MKP-1 repression. In murine mouse models, p38 MAPK kinase was associated with the activation of macrophage reprogramming (216). It has been reported that high dose of FK506, 500 times higher than necessary to suppress CaN, induces short-term p38 MAP kinase activation, reaching its peak at 30 min and subsequently decreasing (215). Furthermore, the application of pharmacological, non-toxic doses of CsA or FK506 effectively inhibits CaN activity, but does not trigger p38 MAPK kinase activation (215). These results strongly suggest that the anti-inflammatory phenotype of macrophages induced by LxVP requires the involvement of p38 MAP kinase. Additionally, the VIVIT peptide also failed to induce p38 activation (215). This unique feature of LxVP distinguishes its action from the properties of other CaN/NFAT inhibitors, such as CsA or FK506. However, before testing this therapy in patients, it would be particularly interesting to unravel the relationship between anti-inflammatory M2 macrophages and macrophages induced by LxVP peptide. Other strategies controlling immune cell function, such as NFAT’s O-linked β-N-acetyl glucosamine modification (217), genetic deletion of soluble epoxide hydrolase (218), or oxygen-ozone therapy that activates immune function and suppresses inflammatory responses through up-regulation of NF-κB, NFAT, and AP-1 signaling (219), should also be given special consideration.

Targeted inhibition of CaN/NFAT signaling, either by naturally occurring compounds or synthetic drugs, may effectively reduce neuroinflammation, thus offering a promising strategy in the treatment of AD (220, 221). However, the translational potential of many CaN inhibitors is markedly limited due to severe adverse effects, including neuro-muscular and renal dysfunction or progressive lymphopenia (222, 223). Despite many advantages over peptide/protein-based drugs, small chemical inhibitors of NFAT have not been widely described. Dipyridamoles can reduce inflammation and cytotoxicity and exert a neuroprotective effect under certain conditions (141, 224). However, their use is not without side effects (225). Another class of small organic molecules, known as INCAs, was initially promising due to their ability to displace VIVIT at low micromolar concentrations, but it turned out to be cytotoxic for certain cell lines when tested. Nonetheless, when administered at non-toxic low micromolar concentrations, INCA-2 and INCA-6 prevented the induction of mRNAs encoding TNFα, interferon-γ, and macrophage inflammatory proteins MIP-1α and MIP-1β (142), as well as some pro-inflammatory cytokines and chemokines (226). Other NFAT inhibitors, such as A-285222, have shown to reduce inflammation, but the experimental data on their use in neuronal or glial cells are very limited (227). Recently, a new hydroxyquinoline derivative, Q134R, has been demonstrated to reduce glial reactivity markers and improve synaptic function, without affecting Aβ load (148). Moreover, Q134R improved cognitive performance and showed no signs of lymphopenia, suggesting its efficacy comparable to CaN inhibitors but with fewer side effects.

## AD-involved receptors signaling through CaN/NFAT

**Figure 6:**
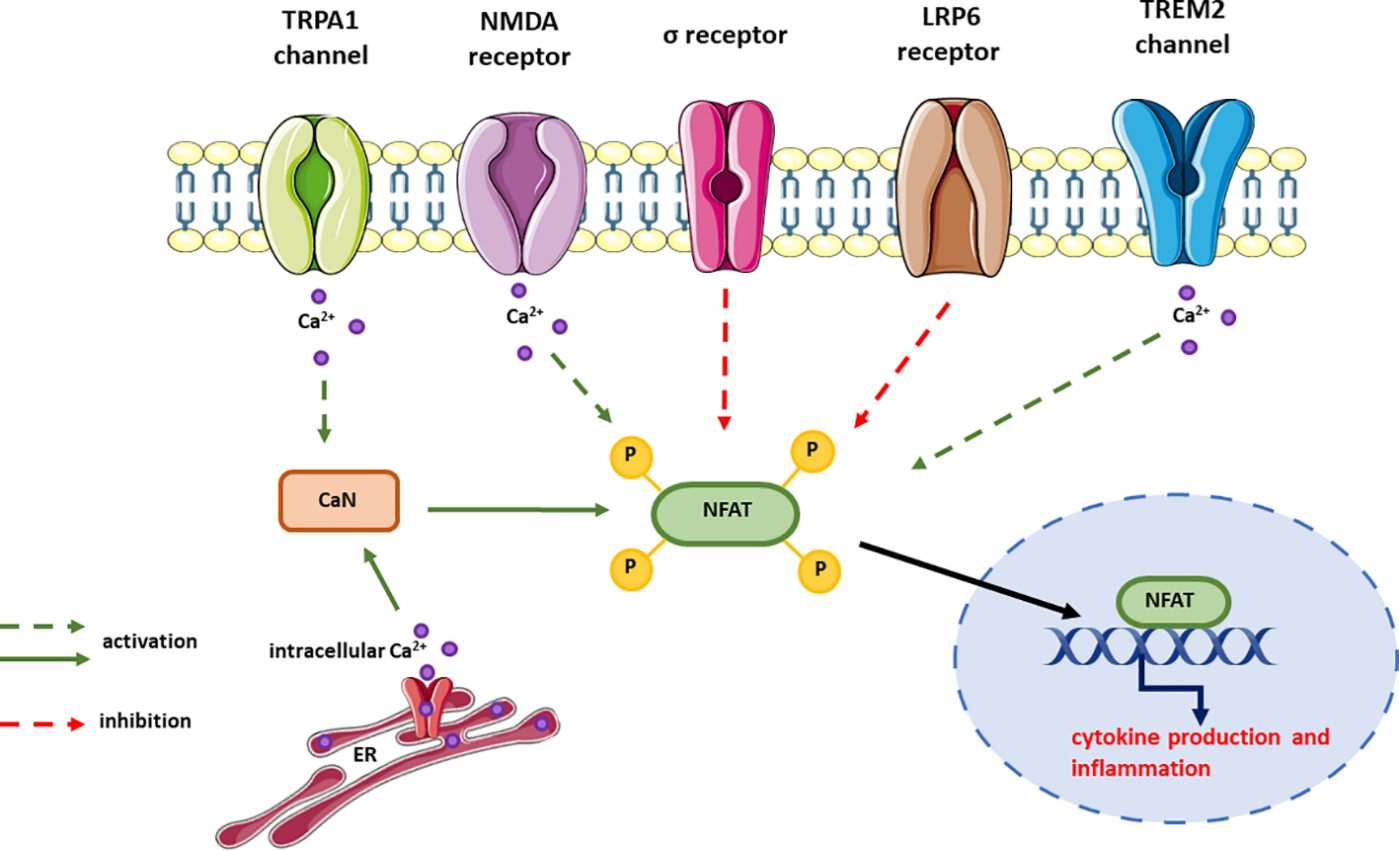
Receptors signaling through CaN/NFAT and their effect on NFAT activation. In most cases, the signaling pathways upstream NFAT but downstream the receptor is unknown and this is expressed as dotted line in the figure. The effect on NFAT activity has been measured either by changes in cytokine expression or release, or the activity of NFAT reporter.

The role of NFAT signaling in AD pathogenesis has been demonstrated in numerous studies, but the upstream signaling leading to CaN/NFAT deregulation remains largely unresolved. Therefore, in this section we discuss how the function of receptors associated with AD affects CaN/NFAT signaling (**Figure 6**).

### Transient receptor potential ankyrin 1

As previously discussed, Aβ-induced Ca^2+^ overload plays a pivotal role in cytokine secretion by activating CaN and its downstream targets, including NFAT and NF-κB (198). However, the intracellular proteins that regulate Aβ-mediated Ca^2+^ influx and drive the inflammatory response are still largely unresolved. One of the strong candidates is the transient receptor potential ankyrin 1 (TRPA1), which is a Ca^2+^-permeable nonselective channel belonging to the TRP channel superfamily. It is widely expressed in the sensory neurons of the dorsal root ganglia, trigeminal ganglia, and nodose ganglia, as well as in hair cells and non-neuronal cells (228). TRPA1 responds to a variety of exogenous irritants and endogenous agonists to mediate inflammation and transmit pain (229–232). Due to these properties, TRPA1 is considered a promising target for novel analgesic and anti-inflammatory drugs. Furthermore, it is involved in temperature sensing and may play a role in the detection of infrared radiation and the avoidance of nociceptive heat (233–238).

Lee and colleagues demonstrated that TRPA1 is upregulated in the hippocampus of APP/PS1 mice (239). In this mouse model, Aβ triggers TRPA1-mediated Ca^2+^ accumulation and hyperexcitability of CA1 hippocampal neurons, suggesting that TRPA1 may function during the early onset of AD (240). Aβ-induced TRPA1-Ca^2+^ signaling has been shown to be a critical event in the activation of CaN/NFAT, leading to cytokine production and inflammation in astrocytes (239, 241). Consistent with this, genetic ablation of TRPA1 channel function in APP/PS1 mouse reduces Aβ-induced activation of CaN, decreases NFAT’s DNA-binding activity in astrocytes, and lowers the levels of pro-inflammatory cytokines IL-1β, IL-6, and IL-4 in the brain. Furthermore, loss of TRPA1 function ameliorates AD progression and improves behavioral performance (239), highlighting the importance of astrocytic TRPA1-Ca^2+^-CaN-NFAT signaling in the inflammatory process and AD progression.

### Sigma receptor

Another channel implicated in AD pathology is the sigma receptor, a Ca^2+^-sensitive chaperone located at the mitochondria-associated endoplasmic reticulum (242). Upon ligand binding, the sigma receptor translocates to the plasma membrane and interacts with various ion channels and G protein-coupled receptors (243). In terms of its mechanism, it operates as more of a signaling modulator than a conventional receptor, and its function encompass the regulation of lipid metabolism, control of both voltage- and non-voltage ion channels, maintenance of intracellular Ca^2+^ homeostasis, modulation of electrical activity, and potentially several other roles (244). Early studies have demonstrated the loss of sigma receptor in the CA1 area of the anterior hippocampus in AD patients, which correlates with damage to CA1 cells (245). The depletion of sigma receptor in the brain is thought to manifest early in the progression of AD, primarily impacting the frontal, temporal, and occipital brain regions (246). The development of preclinical models of AD has allowed researchers to determine the correlation between receptor level and the severity of AD, as well as uncover the neuroprotective properties of sigma receptor agonists (247, 248). For instance, BD-7373 and CB-184, agonists of the sigma-2 receptor subtype, exhibit a potent inhibitory effect on NFAT and NF-κB transcription, leading to decreased expression of TNFα, IL-2, and COX-2 genes, potentially reducing brain inflammation (249). Activation of the sigma-1 receptor, on the other hand, may inhibit γ-secretase activity and reduce Aβ production (250). However, Aβ accumulation and subsequent activation of CaN/NFAT have been shown to induce sigma receptor degradation through NFAT-dependent induction of BIP protein expression, promoting E3 ligase recruitment (251).

### Triggering receptor expressed on myeloid cells 2

TREM2 was initially identified and characterized in human dendritic cells derived from blood monocytes and cultured *in vitro* with the granulocyte macrophage colony stimulating factor and IL-4 (252). It belongs to the immunoglobulin-lectin-like receptor superfamily and plays a role in the development of the AD phenotype (253). TREM2 is predominantly expressed in tissue macrophages, where it can be found both on cell surface and within intracellular compartments. Macrophages expressing TREM2 are located in various sites, including microglia in the central nervous system, specific macrophage subset in the liver, and osteoclast in bone (253). Research has shown that alterations in TREM2 expression affect multiple functions of microglia (254). Increasing TREM2 results in elevated level of chemokine receptors, enhanced cell migration, and improved phagocytosis. Conversely, decreased TREM2 expression inhibits the engulfing of apoptotic cells while increasing the expression of pro-inflammatory cytokines (254).

Characterization of the microglial transcriptome in TREM2-deficient mice has demonstrated the essential role of TREM2 in microglial response during Aβ accumulation (255). TREM2 can be directly modulated by Aβ and other components of Aβ plaques (253). Several studies have reported that TREM2 activation increases intracellular Ca^2+^ level and activates the NFAT reporter system, suggesting that NFAT may transmit signals downstream of TREM2 (252, 256, 257). The precise mechanism of TREM2-dependent NFAT activation in AD is not entirely clear, but a recent study by Zhao et al. revealed that Aβ42 oligomers promote TREM2-DAP12 interaction and SYK kinase phosphorylation, which plays an important role in NFAT signaling (258). Involvement of SYK kinase activating the PLCγ-CaN-NFAT pathway was also suggested by Lessard and colleagues (259). These authors demonstrated that the loss-of-function TREM2 mutation variant R47H, which has been associated with a higher incidence of AD (260), reduced Aβ internalization and NFAT signaling, establishing a direct link between Aβ aggregates, TREM2, and NFAT transcriptional activity. Modulation of NFAT transcriptional activity may also underlie TREM2’s ability to sustain microglial response to Aβ accumulation and influence the production of inflammatory cytokines *in vivo*. This hypothesis is supported by the fact that apoptotic cells transduce the NFAT signal via TREM2, consistent with its function as a sensor of anionic and zwitterionic lipids presented on the surface of neurons exposed to Aβ (261, 262). The accumulation of phosphatidylserine (PS) and phosphatidylethanolamine (PE) on the neuronal cell membrane in the AD mouse brain (263) suggests that PC and PE may transduce signals via TREM2-NFAT (261, 264), potentially enhancing TREM2 activity and promoting a protective phenotype in microglia. Although it remains unclear which TREM2-regulated target genes may be responsible for the increased risk of AD, recent work with engineered TREM2 agonistic antibodies suggests that many of these genes may contain an NFAT response element (265). Activation of TREM2 by dedicated antibodies has been shown to enhance microglia-dependent clearance of Aβ plaques in 5XFAD mice, indicating their potential clinical use in AD treatment. The rationale for strategies aimed at normalizing TREM2 expression/activity in AD treatment is further supported by the latest finding of increased *Trem2* expression in 5XFAD mice (266).

### Imidazoline 2 receptors

Imidazoline 2 receptors (I_2_ receptors) represent a highly heterogenous group of non-adrenergic binding sites characterized by their high-affinity binding to [^3^H]-idazoxan (267). Cellular distribution studies have indicated their presence on the outer mitochondrial membrane, and they have been suggested to potentially serve as novel allosteric binding sites for monoamine oxidase (MAO). Interestingly, another unrelated binding site is brain creatine kinase (268). Numerous biochemical and preclinical investigations, conducted using animal models of brain injury (269–272), suggest that ligands targeting I_2_ receptors exhibit neuroprotective activity, in part by lowering body temperature – an effect known to be beneficial in cerebral ischemia (273). There is also evidence to suggest that I_2_ receptors ligands may mitigate neurodegenerative processes, including cognitive decline, neuroinflammation, and oxidative stress (274).

Indeed, postmortem analysis of brain samples derived from AD patients revealed a higher density of imidazoline 2 receptors (I_2_-IRs), and several pharmacological modulators of I_2_-IRs activity have been successfully tested to reduce AD hallmarks (275–277). The mechanisms underlying the beneficial effects of I_2_-IRs modulation are complex and, so far, largely unknown. Recent reports suggest that neuroprotective mechanisms may be ligand-specific. For instance, LSL6010 alone decreased Aβ plaque formation, tau hyperphosphorylation, and the expression of microglia markers in the 5XFAD mouse model. When administered in combination with donepezil, LSL6010 additionally reduced GFAP reactivity (276). In the same AD murine model, LSL60101 (garsevil) reduced the markers of oxidative stress and decreased the expression of the pro-apoptotic FADD protein in the hippocampus (275). Using the senescence-accelerated mouse prone 8 (SAMP8) model, which is considered a late-onset AD mouse model characterized by tau hyperphosphorylation and altered APP processing (278), Vassilopoulos and colleagues demonstrated that a newly synthesized I_2_-IRs ligand, named B06, reduced the expression of pro-inflammatory cytokines by inhibiting CaN/NFATc1 signaling (274). Therefore, new-generation I_2_-IRs ligands that affect NFAT-dependent transcription hold great neuroprotective potential in AD.

### Low-density lipoprotein receptor-related protein 6

LRP6 belongs to the extended low-density lipoprotein receptor family and serves as a co-receptor in the canonical Wnt signaling. LRP6 is expressed in a widespread manner across human tissues, exhibiting both weak and strong expression patterns in different tissue types (279). Because of its function in Wnt/β-catenin pathway, it is required in the regulation of cell proliferation, specification, migration during development, and it is highly expressed in different types of cancer (280). Furthermore, clinical studies and genomic analysis have confirmed that LRP6 is associated with neurodegenerative diseases including AD (281). Conditional knockout of LRP6 in mice resulted in synaptic dysfunctions, accompanied by cognitive impairments and exacerbated memory deficits in the APP/PSEN1 AD model (282). Interestingly, downregulation of LRP6 expression and LRP6-mediated Wnt signaling were observed in the human brain affected by AD compared to age-matched controls (283). Several possible mechanisms have been proposed to explain how impaired LRP6 downstream signaling may worsen AD pathology. Firstly, LRP6 directly interacts with APP, and its deficiency may promote the amyloidogenic processing of APP, leading to increased endogenous Aβ levels (283). Secondly, LRP6 loss has been shown to increase the number of hippocampal astrocytes and microglia, promoting neuroinflammation through extensive production of pro-inflammatory cytokines (283). LRP6 may also contribute to AD pathology by regulating lipid metabolism, particularly ApoE-containing lipoproteins (284, 285). However, the role of glial and astrocytic LRP6 in the context of AD has not been widely studied. Recent research by Chow and colleagues revealed that LRP6 cell surface retention serves as a bimodal switch for astrocytic fuel metabolism (286). In the absence of LRP6, Wnt signaling activates the non-canonical Ca^2+^‐PKC‐NFAT axis, favoring the utilization of glutamate-derived glutamine and branched chain amino acids over glucose. Increased levels of both NFATc2 and NFATc4 were observed in Wnt‐exposed GFAP‐Lrp6–/– astrocytes, but NFATc2 appears to be the isoform that regulates mitochondrial glutamine metabolism and the acquisition of a reactive phenotype. Depletion of these amino acids from the astrocyte microenvironment may impact synaptic functions and contribute to cognitive and memory deficits. Remodeling of the metabolic framework in astrocytes is an early change associated with late-onset AD (287). In the latest study, non-canonical Wnt5a/CaMKII/NFAT signaling has been shown to participate in the release of inflammatory factors and modulate the activation of microglia (288).

### N-methyl-D-aspartate receptor

Physiologically, NMDARs are central to development of nervous system are involved in a numerous forms of synaptic transmission underlying learning and memory formation (289, 290). In AD, abnormal activation of NMDARs by a glutamate highly released from glial cells stimulates a massive Ca^2+^ influx and aberrant processing of Ca^2+^/CaN signaling, promoting oxidative stress, neuroinflammation and cell death (291–293). Accumulating evidence indicates that function of NMDAR is dysregulated by Aβ and the disruption in Ca^2+^ homeostasis and glutamatergic synaptic transmission may be related to early cognitive deficits observed in AD (294–296). There are multiply potential way by which NMDAR contributes to Aβ pathology: first, Aβ are able to bind NMDAR extracellularly suggesting direct or indirect modulation of the receptor by amyloid β oligomers (297); second, Aβ facilitates NMDAR activation, which controls Aβ production and secretion (298, 299); third, NMDAR may be important mediator in Aβ-induced neurotoxicity (300) and fourth, due to the synaptic and extrasynaptic location, NMDAR may function as a downstream target in Aβ-induced synaptic depression (301). The interaction between Aβ and NMDAR rationalizes the clinical use of memantine, a non-competitive NMDAR antagonist, in combination with acetylcholine inhibitor donepezil, to improve cognitive performance and life quality of patients with moderate to severe AD (302). The beneficial effects of memantine administration may involve reduction in neuroinflammation and overall improvement of brain function although the mechanisms of drug action are not completely understood. The participation of CaN/NFAT cannot be unequivocally excluded as other NMDAR antagonists such as MK-801 are known to abolish NMDAR-dependent NFAT activation (303, 304). Nonetheless, memantine/donepezil did not improve excessive agitation (305) and hippocampal atrophy (306) and the effectiveness of this therapy has been questioned in patients in advanced AD stages (307).

Recently, Turcu et al. developed an optimized, non-cytotoxic, memantine-like NMDAR antagonist, UB-ALT-EV, with high metabolic stability, low micromolar activity and excellent electrophysiological profile as NMDAR blocker (308). The drug rescued defective locomotion and reversed the disrupted chemotaxis behavior in *C. elegans*, which constitutively express human Aβ, suggesting the protection against Aβ toxicity in a manner similar to memantine. Importantly, UB-ALT-EV also enhanced short- and long-term working memory in 5XFAD mice (308). Strikingly, both UB-ALT-EV and memantine normalized CaN protein level, but only UB-ALT-EV, increased NFATc1 phosphorylation, thereby preventing its nuclear translocation (266). Further evaluation of UB-ALT-EV-exerted neuroprotection demonstrated a reduction in the production of several pro-inflammatory cytokines regulated by CaN/NFAT signaling, as well as a decrease in oxidative stress due to enhanced expression of anti-inflammatory mediators in the 5XFAD model (266). These results collectively indicate that UB-ALT-EV’s capacity to reduce gliosis arises from its modulatory effect on NMDAR-dependent Ca^2+^ entry and CaN/NFATc1 downstream signaling.

In mature hippocampal neurons expressing both NMDAR2A and 2B subunits, Aβ-induced hippocampal dysfunctions and ER stress were largely reversed by ifenprodil, an antagonist of NMDAR2B subunits (297). Additionally, ifenprodil prevented the depletion of ER Ca^2+^ content, superoxide generation, and cell death, demonstrating the involvement of NMDAR2B in Aβ neurotoxicity. Considering that NMDAR-dependent NFAT activation and NFAT-mediated transcription are disrupted in primary neurons in the presence of ifenprodil (101), its anti-inflammatory action may involve the normalization of NFAT signaling.

### Other Ca^2+^-permeable receptors

In the *Drosophila* AD model, overexpression of human amyloid precursor protein (APP) induced synaptic hyperexcitability and concurrent upregulation of Ca^2+^-related signaling genes, including Dmca1D (L-type Ca^2+^ channel), CaN, and the inositol 1,4,5-triphosphate receptor (IP3R) (309). Mechanistically, exaggerated Dmca1D expression promoted APP-dependent Ca^2+^ overload, leading to increased CaN activity. This, in turn, triggered NFAT-dependent transcription of IP3R. IP3R is a large-conductance cation channel located in the ER membrane, responsible for cytoplasmic Ca^2+^ increases that control cytoplasmic and mitochondrial processes, thereby regulating cell survival (310, 311). Aberrant Ca^2+^ signaling resulting from IP3R dysregulation has been implicated in several neurodegenerative diseases, including AD, and ER stress-related neuronal injury (312). For instance, IP3R has been previously shown to interact with presenilin mutants causing the familiar form of AD (FAD), leading to its gain-of-function enhancement in an Aβ-independent manner (313, 314). This gain-of-function has been suggested as a key factor behind altered IP3R-mediated Ca^2+^ release in sub-saturating IP3 concentrations, serving as a highly predictive diagnostic feature of AD (315). In light of this, the normalization of IP3R-dependent signaling has been found to restore normal cell function and improve memory in FAD-causing presenilin knock-in mice (316), as well as in triple-transgenic mouse models of FAD (317, 318). Furthermore, Shao et al. demonstrated that NFAT-mediated IP3R upregulation significantly contributes to synaptic downscaling machinery. The restoration of IP3R expression blocked synaptic excitability and miniature excitatory postsynaptic current (mEPSC) frequency (309). Therefore, substantial evidence supports the role of IP3R in contributing to the deregulation of Ca^2+^ homeostasis observed in AD. IP3R dysfunction may drive a series of pathological events leading to disease progression.

IP3R-mediated depletion of ER stores triggers the activation of SOCE (store-operated Ca^2+^ entry) from the extracellular milieu across the plasma membrane, resulting in a subsequent increase in cytosolic Ca^2+^. Altered SOCE-mediated ER store refill is one of the hallmarks of AD, as reduced SOCE has been implicated in synaptic loss and cognitive decline in genetic mouse models as well as in human AD brain samples (319). However, the molecular mechanisms by which IP3R modulates SOCE in AD are not completely understood. A recent study by Sampieri et al. (320) demonstrated that ER depletion induced by G-protein coupled receptor and phospholipase C (PLC) activation stimulates the recruitment of IP3R to the STIM1 protein, which acts as the sensor for ER calcium load. The gradual decrease in STIM1 expression in the medial frontal gyrus of pathologically confirmed AD patients has been linked with disease progression and neurodegeneration mediated by the L-type voltage-dependent Ca^2+^ channel (321, 322). Interestingly, STIM1 expression is sensitive to inflammation (323, 324), suggesting that changes in Ca^2+^ influx through SOCE channels may occur during the early stages of AD development. Using a lipopolysaccharide (LPS)-induced model of AD neuroinflammation, Sun and coworkers (325) demonstrated that SOCE-mediated activation of the PLC/CaN/NFAT pathway up-regulated NADPH oxidase and NOD-like receptor family protein 1 (NLRP1) inflammasome, both playing a pivotal role in oxidative stress and neuronal inflammation. These studies collectively indicate that NFAT activation by pathological Ca^2+^ signals of different origins mediates neurotoxic Aβ effects and contributes to neuronal damage.

## RCAN1 protein- an endogenous control for CaN/NFAT activity

An open question remains regarding the control mechanisms of endogenous CaN/NFAT activity. One of the candidates is the regulator of calcineurin 1 (RCAN1), a small evolutionarily conserved protein that can directly bind to and inhibit CaN activity. RCAN1 has been implicated in various forms of brain degeneration, and its increased expression has been demonstrated in the cortex of patients with AD as well as during normal brain aging (326–329). A conclusive mechanistic explanation for the role of RCAN1 in neuronal death observed in AD has not yet been presented, although several hypotheses have been proposed. Overexpression of RCAN1 is known to induce the caspase-3 mediated apoptotic pathway, which can be blocked by the antioxidant lycopene, suggesting the involvement of oxidative stress (330). In line with this, Sun and coworkers proposed an isoform-specific regulation of RCAN1 by calcium overload, activating CaN/NFAT signaling and exacerbating caspase-3 mediated death (331). According to the study by Jing and colleagues, RCAN1 overexpression disrupts mitochondrial function and promotes apoptosis through the stabilization of adenine nucleotide translocator (ANT1) mRNA and Ca^2+^‐dependent induction of mitochondrial permeability transition pore opening (332). This is supported by the latest study, which shows the inhibition of NFAT and NF-κB transcriptional activity by the RCAN1 RNA aptamer R1SR13, resulting in the attenuation of apoptosis in neurons (333). RCAN1 may also contribute to AD pathology by enhancing N-glycosylation in the ER, thus significantly increasing Aβ production (334). The amyloid proteins and RCAN1 appear to be mutually regulated, as Aβ enhances RCAN1 expression, and RCAN1 reciprocally induces Aβ formation and potentiates its neurotoxicity (334–337). The transcription of RCAN1 can also be activated by NF-κB, a key mediator of brain inflammation in AD (338, 339), and repressed by its own protein in a negative loop. In this regulation, the activation of CaN/NFAT signaling attenuates NF- κB activity, whereas activated NF-κB potentiates NFAT-dependent transcription.

The function of RCAN has been demonstrated to be critical during development and in healthy synapses [reviewed in (340)]. Mice deficient in *rcan1/2* displayed neurological symptoms similar to CaNAβ-null mice, including enhanced locomotor activity and deficits in working memory (341). *Rcan1/2* loss-of-function impaired NFAT activation, suggesting that RCAN may also allow or facilitate CaN-NFAT coupling under certain conditions. This is further supported by a study of Liu and colleagues, which demonstrates that the phosphorylation of RCAN1 at Ser94 and Ser136 by TAK1 (Transforming growth factor beta-activated kinase 1) acts as a switch between inhibitory and permissive RCAN1 functions, thereby enhancing CaN signaling and facilitating NFATc1 nuclear translocation (342). This switch requires the formation of a multimolecular complex involving TAB2 (TGFβ activated kinase 1 binding protein 2) and RCAN1, followed by the recruitment of TAK1, TAB1 and CaN. This mechanism is not observed in *rcan1/2*-deficient mice. As TAK1 and its binding partner TAB1 can be activated by a variety of cytokines, including IL-1β or TNFα (343), RCAN1-dependent modulation of CaN activity may have profound consequences for glial response to pro-inflammatory stimuli. Other studies also confirmed that altering RCAN1 levels either by overexpression or knock-out approaches resulted in memory deficits and impaired synaptic plasticity, both of which are frequently observed in AD patients (344, 345).

Recently, RCAN1 knockdown or overexpression has been linked to age-related deficits in rest-activity and circadian rhythms, characteristic for AD and Down syndrome (346). In the latter, perturbed CaN/NFAT signaling was associated with a higher risk of developing amyloid pathology (347). RCAN1 has also been shown to interact with Dyrk1A, which is considered a candidate protein responsible for AD in the early stages of this disease (348). This interaction allows Dyrk1A-mediated RCAN1 phosphorylation and subsequent CaN inhibition, leading to reduced NFAT activity and enhanced Tau phosphorylation (349). Besides controlling RCAN1, Dyrk1 functions upstream of the GSK-3β kinase, inhibiting NFAT (349), and may directly phosphorylate the NFAT regulatory domain, counterbalancing the effects of CaN-mediated dephosphorylation (75). These observations suggest a direct link between Dyrk1 and RCAN1 in CaN/NFAT signaling, supporting the notion that altered RCAN1/Dyrk1 expression in AD may destabilize the NFAT circuit and contribute to neuropathogenic processes. In line with that, Dyrk1 inhibition by a novel drug, KVN93, reduced neuroinflammation, improved cognitive performance, and decreased Aβ plaque deposition in 5xFAD mice (350). A promising effect toward the amelioration of phenotypic defects observed in AD was also seen with CX-4945 (silmitasertib), which has already undergone clinical trials. Originally developed as a Dyrk1-targeting drug (351), CX-4945 turned out to be a dual Dyrk-1/GSK-3β inhibitor (352) with a strong modulatory effect on AD-related CaN/NFAT signaling and Tau phosphorylation in the mouse hippocampus.

## Concluding remarks

The transcription factors of the NFAT family were originally characterized for their important role in the transcription of cytokine genes and other genes critical for the immune response. The role of NFAT in the neuroinflammatory response in AD is unquestionable. Yet, the reports emerging in recent years suggest that aberrant CaN/NFAT signaling may also play a central deleterious role in brain degeneration, linking amyloid pathology, Ca^2+^ dysregulation, and synapse deterioration. The molecular and phenotypic changes fueled by hyperactive CaN, and cell-specific maladaptive transcriptional programs, may arise early in AD and progress with cognitive decline. These deleterious changes in transcriptional control are observed both in neurons and astrocytes and likely involve the NFAT component. The numerous studies in transgenic animal AD models showing beneficial effects of CaN and/or NFAT inhibitors are consistent with this hypothesis. Further work is needed to better characterize the upstream signals leading to CaN/NFAT overactivation in AD and dissect the actions of this pathway on different transcriptional regulatory mechanisms. Moreover, it would be desirable to unravel how these changes drive synaptic malfunction and how targeted molecular interventions may help slow down cognitive decline.

## Author contributions

JM: Conceptualization, Resources, Software, Visualization, Writing – original draft, Writing – review & editing. ML: Software, Supervision, Visualization, Writing – original draft, Writing – review & editing. TB: Conceptualization, Funding acquisition, Resources, Supervision, Writing – original draft, Writing – review & editing.
